# Primary Angiitis of the Center Nervous System: A Clinical Challenge Diagnosed Postmortem

**DOI:** 10.1155/2017/3870753

**Published:** 2017-07-05

**Authors:** Bayan Al Share, Ali Zakaria, Evan Hiner, Ziyad Iskenderian, Nader Warra

**Affiliations:** ^1^Department of Internal Medicine, Providence-Providence Park Hospital, Michigan State University College of Human Medicine, Southfield, MI, USA; ^2^Division of Neurology, Providence-Providence Park Hospital, Michigan State University College of Human Medicine, Southfield, MI, USA

## Abstract

Primary angiitis of the central nervous system (PACNS) is a rare vasculitis involving medium and small blood vessels of the brain, spinal cord, and meninges, without systemic involvement. The diffuse and patchy nature of its pathology is reflected by a wide spectrum of nonspecific clinical symptoms. Diagnosis is challenging due to lack of defined clinical criteria or specific imaging findings. Specific workup should be done only after exclusion of other etiologies, including infectious, neoplastic, toxic, and other vascular etiologies including systemic vasculitis. Given the fact that it is a patchy disease with 25% of the biopsies being falsely negative, treating physician should have a high index of suspicion despite negative initial neurovascular imaging and biopsy results. Once diagnosed, early treatment with immunosuppressive therapy is essential to avoid permanent neurologic damage. Herein, we are reporting a case of 66-year-old female patient who presented with insidious onset right-sided frontal headache. Her hospital course progressively worsened and family decision based on her wishes was to refer her to hospice and comfort care. Despite an extensive workup with advanced imaging techniques, no diagnosis was established until postmortem autopsy and histopathology confirmed primary angiitis of the central nervous system.

## 1. Introduction

Primary angiitis of the central nervous system (PACNS) is a rare vasculitis involving medium and small blood vessels of the brain, spinal cord, and meninges, without systemic involvement. The disease considered a diagnostic challenge since there are no defined clinical criteria or specific imaging findings. Herein, we present a case of PACNS that was a diagnostic dilemma till postmortem brain biopsy confirmed it.

## 2. Case Presentation

A 66-year-old female with a previous medical history of hypertension, type II diabetes mellitus, and hyperlipidemia presented to the emergency department with right-sided frontal, throbbing, and nonradiating headache of insidious onset that awaken her from sleep and progressively worsened overnight. The pain was not relieved with acetaminophen or NSAIDs, and it was associated with nausea and multiple episodes of vomiting. She denied dizziness, impaired consciousness, vision changes, fever, chills, recent sick contact, and travel. She had no previous history of similar headaches and no history of migraine. On physical examination she was awake but confused, in severe pain holding her head because of the headache. Her vital signs were temperature 36.9°C, BP 220/86 mmHg, PR 90 bpm, and RR 18 bmp. Neurological examination revealed normal cranial nerves (I–XII), no neck rigidity, negative Brudzinski and Kernig's signs, power 5/5 over upper and lower extremities, intact sensation, and deep tendon reflexes II/IV.

An urgent computed tomography (CT) scan of brain was ordered which revealed no intracranial bleeding or mass occupying lesions. However, after the CT scan, she had a generalized tonic-clonic seizure that was controlled with one dose of lorazepam. A lumbar puncture was done and revealed: no xanthochromia, 912 red blood cells (RBCs), 3 white blood cells (WBCs) with no polymorphonuclear cells, 43 mg/dL protein, and 87 ng/dL glucose (serum glucose 200 mg/dL), cerebrospinal fluid gram stain and culture were negative. CT angiogram revealed 30% stenosis of the right internal carotid artery and no other abnormalities.

She had a second seizure with apneic episodes, so an elective endotracheal intubation was performed and she was transferred to the neurosurgical intensive care unit (NSICU). A magnetic resonance imaging (MRI) of the brain revealed cortical restricted diffusion edema as well as sulcal FLAIR hyperintensity and diffuse leptomeningeal enhancement, which had been followed by a magnetic resonance angiogram (MRA) of the brain that showed faint opacification of left transverse sinuses, and it was difficult to exclude nonocclusive or chronic thrombosis. Results were correlated with a CT angiography that revealed no occlusion or thrombosis. ESR was 45 mm/hr, CRP was 106 mg/L, and antibody screen including ANA, rheumatoid factor, C-ANCA, and P-ANCA were negative.

**Figure 1:**
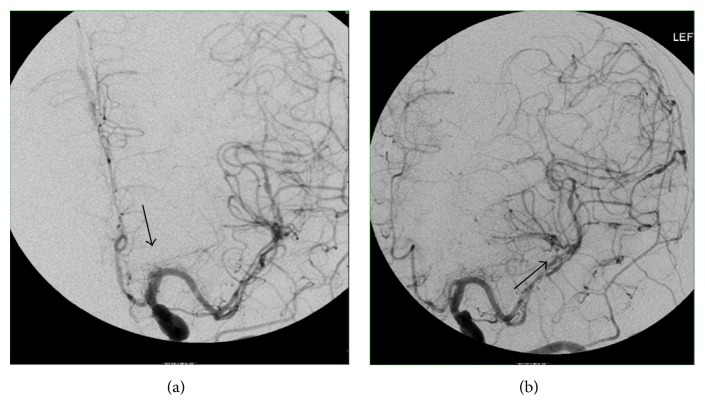
Conventional cerebral angiography revealed (a) M1 segments of the middle cerebral artery and (b) M2 segments of the middle cerebral artery with classic beading pattern of constrictions indicative of cerebral vasculitis (black arrows).

Upon weaning her from sedation, she was not responsive and she failed to obey commands. A repeated CT scan of the head revealed an acute ischemic infarct of the right occipital and posterior temporal lobes. A conventional angiography was performed and revealed diffuse cerebral vascular beading pattern of constriction involving vasculature of both hemispheres, a pattern indicative of diffuse vasculitis (Figure 1). She was started on methylprednisone 1 gram intravenously. A brain biopsy (from cerebral cortex, meninges and meningeal vessels, and right superficial temporal artery) was performed and pathology report revealed no evidence of vasculitis.

**Figure 2:**
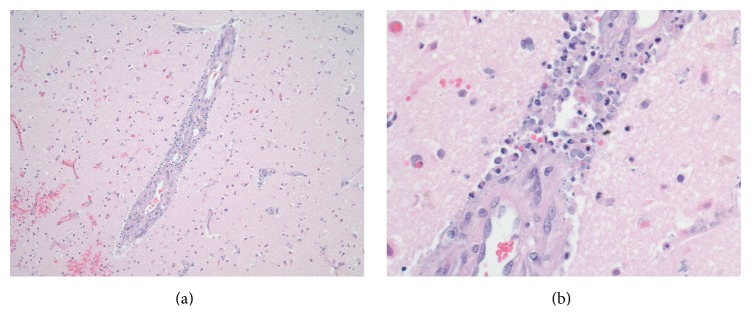
Histopathology of the cerebral cortex containing medium sized blood vessel. (a) H&E stain ×40 reveals acute inflammation concentrated in vessel wall. (b) H&E stain ×400 reveals acute necrotizing vasculitis; dense neutrophilic inflammatory exudate; and necrosis in vessel wall.

The patient's medical situation progressively worsened and follow-up CT scan of the brain revealed development of new hypodensities noted in the right posterior occipitoparietal, left occipital, and bilateral frontal regions. At this point, a family meeting was held and according to patient's wishes, the decision was made for comfort measures and terminal weaning from the ventilator. An autopsy exam was performed, with brain tissue histopathology significant for scattered areas of vasculitis involving arterioles and numerous relatively recent infarcts (Figure 2).

## 3. Discussion

Primary angiitis of the central nervous system (PACNS) is a rare vasculitis involving medium and small blood vessels of the brain, spinal cord, and meninges, without systemic involvement, causing a heterogeneous disease with different clinical subsets of unknown etiology and pathogenesis. It affects patients of all ages but peaks around 50 years of age. The reported annual incidence rate is 2.4 cases per 1,000,000 person-year with 2 : 1 male to female ratio [1].

The diffuse and patchy nature of its pathology is reflected by a wide spectrum of nonspecific clinical symptoms that are typically subacute and proceeded by long prodromal period [2, 3]. Headache is the most common symptom, reported by about 60% of patients [1, 4]. Other symptoms include those of stroke, cognitive impairment, and transient ischemic attacks [1, 5] and less commonly cranial neuropathies, ataxia, seizures, chronic meningitis with no known infections or neoplastic etiology, and coma. Symptoms due to spinal cord involvement include painful myelopathy, motor weakness, and sensory findings that can present alone or coincide with those of brain involvement [6]. However; marked constitutional symptoms and weight loss are not typical of PACNS and should raise suspicion of systemic illness [7].

The diagnosis of PACNS is challenging since there are no defined clinical criteria or specific imaging findings. Therefore, approach for diagnosis varies with the clinical setting. Specific workup should be done only after excluding other conditions, such as reversible cerebral vasoconstriction syndrome (RCVS), infections, neoplastic processes, drug or toxin exposures, hypercoagulable state, and vasospastic processes, as well as excluding systemic vasculitis as a cause [7]. Workup to rule out infectious etiology and measuring acute phase reactants and antibodies to rule out systemic vasculitis are essential in approaching these patients, given that PACNS-unlike systemic vasculitis- is negative to antibodies and lacks elevation in acute phase reactants [8, 9].

However, some infectious pathogens such as Varicella Zoster virus, West Nile virus,* Mycoplasma gallisepticum*, and Human Immunodeficiency virus have been proposed as etiologies for PACNS [10–12]. Therefore, CSF analysis is essential as a part of diagnostic workup, as in over 90% of cases, it revealed an evidence for aseptic meningitis [8].

Neuroimaging is essential in diagnosing PACNS. Magnetic resonance imaging (MRI/MRA) is more sensitive (75–100%) than computed tomography (CT) scans for detection of vascular changes needed for diagnosis [8]. In general, normal brain MRI combined with negative CSF analysis has a high negative productive value for diagnosis of PACNS [13]. On MRI, findings include infarcts, with or without contrast enhancement, that are often bilateral in cortex, deep, white matter or leptomeninges. Computed tomography (CT) angiography is more sensitive than MRA in detecting vascular irregularities but catheter-based angiography is far more sensitive, though its estimated sensitivity in the literature is between 27 and 90% limited by caliber of the vessel, being most sensitive for disease of larger vessels. Also, angiography has specificity of 26% [14]. Classic angiography findings in PACNS include segmental narrowing and beading appearance of small and medium vessels and circumferential or eccentric vessel irregularities [15]. An unusual presentation on angiography is an avascular mass effect.

The gold standard for diagnosis is the histologic examination, though it requires a highly invasive procedure. PACNS is a patchy disease since as many as 25% of the biopsies are falsely negative [16]. Histopathologic findings include granulomatous angiitis with Langerhans' giant cells, necrotizing vasculitis, or lymphocytic vasculitis.

Upon diagnosis, early treatment is essential to avoid permanent neurologic damage. In some cases, it is wise to start empiric glucocorticoids while the full workup is completed. Patients with confirmed granulomatous inflammation on pathology should be started on immunosuppressive therapy that contains a combination of glucocorticoids and cyclophosphamide [2, 17]. Rituximab can be used in case of intolerance for cyclophosphamide. Immunosuppression has been associated with success in CNS vasculitis; however, none of the studies were conducted in patients with PACNS [1].

Monitoring disease activity is another challenge due to difficulty differentiating irreversible target-organ damage from treatment-resistant disease on neuroimaging. Therefore, response to treatment for some patients may only be indicated by symptomatic improvement rather than resolution of imaging findings. But in general, a follow-up MR should be obtained four to six weeks after beginning treatment, then every three to six months throughout therapy, and subsequently according to the evolution of the disease [9, 18]. Failure to respond to therapy should raise suspicion for an alternative diagnosis before additional treatment with an alternative immunosuppressive drug is used.

## 4. Conclusion

Primary angiitis of the central nervous system (PACNS) is a rare vasculitis that lacks defined clinical criteria for diagnosis which leads to variable diagnostic approaches when suspected. Given the patchy involvement pattern of the disease with 25% of the biopsies being falsely negative, treating physician should have a high index of suspicion despite negative initial neurovascular imaging and biopsy results.
